# Effect of constant collision mean free time on the boundary layer of the active collisional warm plasma

**DOI:** 10.1038/s41598-021-97750-1

**Published:** 2021-09-15

**Authors:** Mansour Khoram, S. Farhad Masoudi

**Affiliations:** 1 Department of Basic Sciences and Engineering, Borujerd Branch, Islamic Azad University, P. O. Box 6915136111, Borujerd, Iran; 2 Department of Physics, K. N. Toosi University of Technology, P. O. Box 15875-4416, Tehran, Iran

**Keywords:** Atomic and molecular physics, Plasma physics

## Abstract

The plasma boundary layer is analyzed for a plasma in contact with a conducting plain surface where the ion temperature is comparable with the electron temperature and the plasma pressure is sufficiently high. The variations of electrical potential from the plasma-presheath boundary to the wall is studied using the fluidal formalism of plasma in three approaches; plasma and sheath asymptotic solutions and full solution. In the full solution approach, fluidal equations lead to a singularity when the ion velocity reaches the ion thermal speed. It is shown that removing the singularity causes a well-defined eigenvalue problem and leads to smooth solutions for the model equations. Some of the applicable aspects such as the floating velocity and density of ions, the floating electrical potential and an estimation of the floating thickness of the boundary layer are obtained. The dependency of these quantities on the ionization degree, the ion temperature and ion-neutral collision is examined too.

## Introduction

There is a considerable attention to the correct cognition of the positive column in plasmas, especially near walls or electrodes^1^. At the boundary layer of plasma where a plasma contacts a negatively biased or floating electrode, an electric field is emerged between the plasma edge and that electrode. The electric field attracts the positive ions toward the wall and repels the electrons toward the plasma. The currents of ion and electron into the wall are eventually maintained according to the wall electric potential in the steady state.

One can assume the plasma boundary layer as a sequence of the neutral presheath next to the plasma and the ion rich sheath next to the wall. The both regions contact each other at a critical point so called the sheath edge in which the ions need to attain at least the Bohm velocity in order to enter the sheath. Accelerating the ions to the Bohm velocity, and even more than that, takes place in the presheath. Generally, the neutral presheath is responsible for the ions flux while the function of the non-neutral sheath is to prepare the required ions energy on the surface. Typically, the sheath is very thin, while the presheath is too wide in the real scale. Then, it is necessary to formulate the plasma boundary layer in different scales if one needs to study the content correctly^2^.

Since the presheath and sheath regions in their own scales show completely different behavior in the limiting case $\epsilon = \lambda_{D} /L \to 0$, it is necessary to match the two regions in an intervene scale so called the intermediate scale. Here, $\lambda_{D}$ is the electron Debye length and is known as the sheath scale and $L$ is a competing characteristic length that can be the presheath width or the ion-neutral collision mean free path^3^. Indeed, the intermediate scale is a bridge to cross the sheath edge singularity and to patch the presheath and sheath asymptotic solutions for some finite values of $\epsilon$.

Unfortunately, the validity of the intermediate scale analysis is limited to a very narrow vicinity of the sheath edge. When the analytical approximation expression, which is used to match uniformly all three asymptotic solutions of the plasma, is constructed and compared with the numerical full solutions of equations, it is exhibited that the validity of the matching method is violated for $\epsilon > 10^{ - 4}$^2^. So, it is necessary to find the numerical solution of the plasma boundary layer equations in the whole region from the plasma-presheath interface (or presheath edge) to the wall.

The energy and flux of ions attained through the boundary layer control the physical processes at surfaces that confine the plasma. These plasma-surface interactions are important in many fields including plasma processing^4,5^, plasma diagnostics^6^, plasma ion sources^7,8^ and fusion devices^9^. Both ion-neutral elastic collision and ion temperature in the boundary layer of plasma can significantly affect the ion impact energy and flux on the surface, so it is worthwhile to include them in the analysis of the plasma boundary layer.

Many authors have studied the structure and behavior of plasma boundary layer in warm collisional electropositive^10–29^ and electronegative^29–38^ plasmas. They have used one-dimensional fluidal models, in which the positive ions are treated with the continuity and momentum transfer equations, while electron and negative ions are assumed to fulfill the Boltzmann relation. These equations completed by the Poisson equation for the electric potential involve a singular point if the ion pressure (temperature) is also taken into account. This singularity is located at the ion thermal speed $c_{th} = \sqrt {k_{B} T_{i} /m_{i} }$ in the ion isothermal approximate, where $k_{B}$, $T_{i}$ and $m_{i}$ are the Boltzmann constant, ion temperature and ion mass respectively. The singularity should be removed and it is essential to find a smooth transition from the presheath edge to the wall surface.

There are several studies to find the ion temperature, magnetic field and collision effects on the sheath criterion and structure^13–26^. Assuming a small ion temperature in some studies, the singularity has been removed and the set of singular equations has been solved by Taylor series expansions around the singular point with the ratio $T_{i} /T_{e}$ as the expansion parameter in which, $T_{e}$ is the electron temperature^10,11,28,34,35^. There are another studies in which the fluid equations have been solved numerically just after the singular point for some small ion temperature^37,38^. They found the essential initial values at the singular point for solving the equations. Gyergyek and Kovacic^27^, and Valentini and Kaiser^39^ have used the solution of the one-fluid model (plasma solution) in an interval around the singularity point to find a numerical smooth solution to cross the singular point in the two-fluid model of plasma. Khoram has used an approximate method near the singular point to cross it in a collision free electropositive plasma with constant ion temperature^29^.

The present paper investigates the boundary layer of thermal electropositive plasma from the presheath edge, with quasi-neutrality condition, to the floating wall, when the ions temperature is comparable to the electrons temperature and there is an elastic ion-neutral collision with constant ion mean free time. This is the simpler model for collisional momentum transfer in comparison with that of constant ion mean free path which is more complicated and is used in high pressure plasmas. In this sense, we present an active plasma in a two-fluidal model in which the equations are singular when the ions are not cold. To remove this thermal singularity and find a smooth solution for the equations, we use a classical method so called the Hopital’s rule. This enables us to analyze how the ion temperature and collision parameter affect on the boundary layer structure and especially on the floating variables.

Following the introduction, the plasma model and its equations are presented in “Mathematical modeling of plasma” section. In “Asymptotic limit of plasma” section and “Asymptotic limit of sheath” section, the asymptotic limits of plasma and sheath are introduced, respectively. In addition, these two sections contain the analytical solutions of the plasma equations and the numerical solutions of the sheath equations. The plasma full equations in the plasma scale are presented in “Full solution of the eigenvalue problem” section. In order to solve the full equations of plasma, the proper boundary conditions and the method of removing the singularity in these equations are introduced in "Methods and boundary conditions" section. Finally, the boundary layer structure, the floating variables and the sheath thickness according to the ion temperature, collision parameter and smallness parameter (ionization degree) are extracted and investigated in “Results and discussion” section. The paper is concluded in “Summary and conclusions” section.

## Mathematical modeling of plasma

The positive column of warm plasmas containing neutral atoms, electrons and singly-charged positive ions is studied near the wall region by assuming the plane geometry of conducting walls. Ions and electrons are created in the plasma through single-stage collisions between neutral atoms and energetic electrons. Then, they stream to the wall, recombine there and appear as the neutral atoms in the plasma again.

The conducting walls are charged and their electric potential decreases with respect to the plasma center. The arisen electric force, together with the thermal, collisional and inertial forces restricts the ions and electrons movement towards the wall. The steady state continuity and momentum transfer equations for ions and electrons, with along the Poisson equation comprise the governing equations as follows

1 $$\nabla \cdot \left( {n_{i} {\mathbf{v}}_{i} } \right) = \nu_{I} n_{e} ,$$

2 $$m_{i} \nabla \cdot \left( {n_{i} {\mathbf{v}}_{i} {\mathbf{v}}_{i} } \right) = en_{i} {\mathbf{E}} - \frac{{m_{i} n_{i} {\mathbf{v}}_{i} }}{{\tau_{ec} }} - \nabla \cdot {\mathbf{P}}_{i} ,$$

3 $$\nabla \cdot {\mathbf{E}} = \frac{e}{{\epsilon_{0} }}\left( {n_{i} - n_{e} } \right),$$

4 $$0 = - en_{e} {\mathbf{E}} - \nabla \cdot {\mathbf{P}}_{e} ,$$

where $n_{i}$, ${\mathbf{v}}_{i}$ and $m_{i}$ are the ion density, velocity and mass respectively, ${\mathbf{P}}_{i}$ and ${\mathbf{P}}_{e}$ are the ion and electron stress tensors respectively, and ${\mathbf{E}} = - \nabla V$ is the electric field with $V$ as the electric potential. Also, $n_{e}$ is the electron density, $\nu_{I}$ stands for the *ionization* frequency, $\tau_{ec}$ is the mean free time of the ion-neutral *elastic* collision, $e$ is the elementary charge and $\epsilon_{0}$ is the vacuum permittivity. The inertial and collisional forces have been neglected in Eq. (4) since electrons are much lighter than ions.

**Figure 1:**
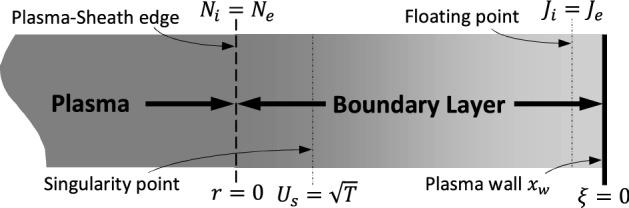
Schematic graph of the simulation zones in the plasma boundary layer.

It is assumed that there is homogeneity parallel to the wall, so the space variations are found just in direction perpendicular to the wall ($x$-axis). Out of symmetrical configuration of plasma around the central plane $x = 0$, the studying of plasma will be done in the half space $x \ge 0$. A schematic diagram of the simulation zones in the plasma boundary layer is shown in Fig. 1.

The general stress force $- \nabla \cdot {\mathbf{P}}$ is reduced to $- {\text{d}}P/{\text{d}}x = - k_{B} T\left( {{\text{d}}n/{\text{d}}x} \right)$ for the both of ions and electrons in the simplest case, i.e. isotropic Maxwellian plasmas with the ion and electron isothermal flows^33^. Here, $T$ could be replaced by the absolute temperature of ions $T_{i}$ or electrons $T_{e}$. Also, the collision mean free time is truly given by $\tau_{ec} = 1/n_{n} \sigma_{s} v_{s}$ in which $n_{n}$ is the number density of inert *neutral* atoms and $\sigma_{s}$ is the momentum transfer cross section for the ions moving with the ion *acoustic* speed $c_{s} = \sqrt {k_{B} T_{e} /m_{i} }$^26^. With these assumptions, it is straightforward to rewrite (1)–(4) in the normalized form as

5 $$\frac{{{\text{d}}\left( {IU} \right)}}{{{\text{d}}r}} = N,$$

6 $$IU\frac{{{\text{d}}U}}{{{\text{d}}r}} = H - \tau \frac{{{\text{d}}I}}{{{\text{d}}r}},$$

7 $$\epsilon^{2} \frac{{{\text{d}}F}}{{{\text{d}}r}} = I - N,$$

8 $$\frac{{{\text{d}}N}}{{{\text{d}}r}} = - NF.$$

Using Eqs. (5) and (6) results in;

9 $$\frac{{{\text{d}}I}}{{{\text{d}}r}} = \frac{Q}{R},$$

10 $$\frac{{{\text{d}}U}}{{{\text{d}}r}} = \frac{S}{R},$$

where

11 $$H = FI - \left( {N + \alpha I} \right)U,\quad Q = NU - H,\quad S = \frac{UH - \tau N}{I}, \quad R = U^{2} - \tau .$$

In Eqs. (5)–(8) and (11), the following dimensionless parameters and variables have been utilized;

$$\begin{aligned} & \alpha = \frac{1}{{\tau_{ec} \nu_{I} }},\quad \lambda_{s} = \frac{{v_{s} }}{{\nu_{I} }},\quad \epsilon = \frac{{\lambda_{D} }}{{\lambda_{s} }},\quad \tau = \frac{{T_{i} }}{{T_{e} }},\quad \phi_{0} = \frac{{k_{B} T_{e} }}{e},\quad E_{0} = \frac{{\phi_{0} }}{{\lambda_{s} }}, \\ & r = \frac{x}{{\lambda_{s} }},\quad U = \frac{{v_{x} }}{{v_{s} }},\quad F = \frac{{E_{x} }}{{E_{0} }},\quad \phi = - \frac{V}{{\phi_{0} }},\quad I = \frac{{n_{i} }}{{n_{0} }},\quad N = \frac{{n_{e} }}{{n_{0} }} \\ \end{aligned}$$

in which, the dimensionless parameters $\tau$, $\alpha$, and $\epsilon$ are the temperature, collision and smallness parameters, respectively, $\lambda_{D} = \sqrt {\epsilon_{0} k_{B} T_{e} /n_{0} e^{2} }$ defines the Debye length at the plasma center, and $F = {\text{d}}\phi /{\text{d}}r$ is the normalized electric field. Then, one can easily conclude that $N = {\text{exp }}\left( { - \phi } \right)$ from Eq. (8).

Applying $\epsilon \to 0$ in the Poisson’s equation, Eq. (7) results to $I = N = {\text{exp }}\left( { - \phi } \right)$, that is called the plasma approximation. In the plasma approximation, there is no sheath in front of the wall. However, it is sometimes necessary to introduce the sheath region including a positive space charge even in the asymptotic limit $\epsilon \to 0$. In order to study the sheath region, it is needed to use the new space coordinate $\xi = \left( {x - x_{w} } \right)/\lambda_{D} = \left( {r - r_{w} } \right)/\epsilon$ in which, $x_{w}$ (or $r_{w}$) is the location of the wall selected as the origin of the new coordinate. Equations (5)–(7) in the new coordinate space are transformed to

12 $$\frac{{{\text{d}}\left( {IU} \right)}}{{{\text{d}}\xi }} = \epsilon N,$$

13 $$U\frac{{{\text{d}}U}}{{{\text{d}}\xi }} = \frac{{{\text{d}}\phi }}{{{\text{d}}\xi }} - \epsilon \left( {\frac{N}{I} + \alpha } \right)U - \frac{\tau }{I}\frac{{{\text{d}}I}}{{{\text{d}}\xi }},$$

14 $$\frac{{{\text{d}}^{2} \phi }}{{{\text{d}}\xi^{2} }} = I - N,$$

knowing that $N = {\text{exp }}\left( { - \phi } \right)$. Although, there is an explicit difference between them under the asymptotic condition $\epsilon \to 0$, there is no difference between the set Eqs. (5)–(7) and (12)–(14) for $\epsilon > 0$.

## Asymptotic limit of plasma

The plasma asymptotic limit means applying the condition $\epsilon \to 0$ in the plasma approach equations. In this limiting case, Eqs. (5)–(7) turns to

15 $$\frac{{{\text{d}}\phi_{p} }}{{{\text{d}}r}} = \frac{{\left( {2 + \alpha } \right)U_{p} }}{{1 + \tau - U_{p}^{2} }} ,$$

16 $$\frac{{{\text{d}}U_{p} }}{{{\text{d}}r}} = \frac{{1 + \tau + \left( {1 + \alpha } \right)U_{p}^{2} }}{{1 + \tau - U_{p}^{2} }} ,$$

17 $$I_{p} = N_{p} = {\text{exp}}\left( { - \phi_{p} } \right),$$

where the subscription “*p*” refers to the *plasma* asymptotic limit. Equations (15) and (16) can be solved analytically to get

18-a $$\phi_{p} = \frac{2 + \alpha }{{2\left( {1 + \alpha } \right)}}{\text{ln}}\left( {1 + \frac{1 + \alpha }{{1 + \tau }}U_{p}^{2} } \right) ,$$

18-b $$r_{p} = \frac{1}{1 + \alpha }\left[ {\sqrt {\frac{1 + \tau }{{1 + \alpha }}} \left( {2 + \alpha } \right){\text{arctan}}\left( {\sqrt {\frac{1 + \alpha }{{1 + \tau }}} U_{p} } \right) - U_{p} } \right]$$

18-c $$I_{p} = N_{p} = {\text{exp}}\left( { - \phi_{p} } \right) = \left( {\frac{1 + \tau }{{1 + \tau + \left( {1 + \alpha } \right)U_{p}^{2} }}} \right)^{{\left( {2 + \alpha } \right)/2\left( {1 + \alpha } \right)}}$$

for $0 \le U_{p} \le \sqrt {1 + \tau }$. However these universal functions are not practical and one needs to find the numerical full solutions in more applicable situations for $\epsilon > 0$, yet they represent some primary knowledge about the collision and temperature effects in the real plasmas. These functions are transferred to

19-a $$\phi_{p} = {\text{ln}}\left( {1 + \frac{1}{1 + \tau }U_{p}^{2} } \right)$$

19-b $$r_{p} = \left[ {2\sqrt {1 + \tau } {\text{ arctan}}\left( {\frac{{U_{p} }}{{\sqrt {1 + \tau } }}} \right) - U_{p} } \right]$$

19-c $$I_{p} = N_{p} = {\text{exp}}\left( { - \phi_{p} } \right) = \frac{1 + \tau }{{1 + \tau + U_{p}^{2} }} ,$$

in the collisionless warm plasmas $\left( {\alpha = 0} \right)$^29^, and are simplified to

20-a $$\phi_{p} = {\text{ln}}\left( {1 + U_{p}^{2} } \right) ,$$

20-b $$r_{p} = 2{\text{ arctan }}U_{p} - U_{p} ,$$

20-c $$N_{p} = \frac{1}{{1 + U_{p}^{2} }}.$$

in the collisionless cold plasmas $(\tau = 0$ and $\alpha = 0)$^2^.

Apparently there is a singular point in Eqs. (15) and (16) which is introduced by $U_{b} = \sqrt {1 + \tau } = U_{B}$ and is called the Bohm criterion. This singularity point represents the plasma-sheath boundary where the breakdown of the quasi-neutrality is commenced. The other variables at the plasma-sheath boundary can be found using Eq. (18) as follows;

21-a $$\phi_{b} = \frac{2 + \alpha }{{2\left( {1 + \alpha } \right)}}{\text{ln}}\left( {2 + \alpha } \right)$$

21-b $$r_{b} = \frac{{\sqrt {1 + \tau } }}{1 + \alpha }\left[ {\frac{{\left( {2 + \alpha } \right)}}{{\sqrt {1 + \alpha } }}{\text{arctan}}\left( {\sqrt {1 + \alpha } } \right) - 1} \right]$$

21-c $$I_{b} = N_{b} = {\text{exp}}\left( { - \phi_{b} } \right) = \left( {\frac{1}{2 + \alpha }} \right)^{{\left( {2 + \alpha } \right)/2\left( {1 + \alpha } \right)}} .$$

By the way, these marginal variables in the collisionless cold plasmas are reduced to the well known quantities $U_{b} = 1$, $\phi_{b} = {\text{ln }}2$, $r_{b} = \pi /2 - 1$, and $I_{b} = N_{b} = 1/2$^2,24,39^.

## Asymptotic Limit of Sheath

The sheath asymptotic limit means applying the condition $\epsilon \to 0$ in the sheath approach equations. Using this condition in Eqs. (12)–(14) turns them to

22 $$I_{s} U_{s} = I_{b} U_{b} ,$$

23 $$U_{s}^{2} - U_{b}^{2} = 2\left[ {\phi_{s} - \phi_{b} - \tau {\text{ln}}\left( {\frac{{I_{s} }}{{I_{b} }}} \right)} \right] ,$$

24 $$\frac{{{\text{d}}^{2} \phi_{s} }}{{{\text{d}}\xi^{2} }} = I_{s} - N_{s} ,$$

where subscription ‘*s’* refers to the *sheath* asymptotic solution. Combining Eqs. (22) and (23) results in

25 $$I_{s} = I_{b} \left[ {1 + \frac{{2\psi_{s} }}{{U_{b}^{2} }} + \frac{2\tau }{{U_{b}^{2} }}{\text{ln}}\left( {\frac{{I_{b} }}{{I_{s} }}} \right)} \right]^{ - 1/2}$$

with $\psi_{s} = \phi_{s} - \phi_{b}$ as the relative electric potential. Equation (25) in the cold plasmas reduces to the well-known relation^29^

26 $$I_{s} = I_{b} \left( {1 + \frac{{2\psi_{s} }}{{U_{b}^{2} }}} \right)^{ - 1/2} .$$

In order to find the sheath approach solutions, it is needed to solve the second-order differential equation (ٍ24) from the plasma-sheath boundary to the wall. To find the essential boundary conditions, it is necessary to expand $I_{s}$ and $N_{s}$ in powers of $\psi_{s}$ at the plasma-sheath border (where $\psi_{s} \to 0$) as follows

27 $$I_{s} \approx I_{b} \left[ {1 - \frac{1}{{U_{b}^{2} }}\left( {1 + \frac{\tau }{{U_{b}^{2} }}} \right)\psi_{s} + \frac{1}{{U_{b}^{4} }}\left( {\frac{3}{2} + \frac{4\tau }{{U_{b}^{2} }} + \frac{{3\tau^{2} }}{{2U_{b}^{4} }}} \right)\psi_{s}^{2} + O\left( {\psi_{s}^{3} } \right)} \right] ,$$

28 $$N_{s} = N_{b} {\text{ exp}}\left( { - \psi_{s} } \right) \approx N_{b} \left[ {1 - \psi_{s} + \frac{1}{2}\psi_{s}^{2} + O\left( {\psi_{s}^{3} } \right)} \right].$$

Using above approximations in (24) results in

29 $$\frac{{{\text{d}}^{2} \psi_{s} }}{{{\text{d}}\xi^{2} }} \approx I_{b} \left[ {1 - \frac{1}{{U_{b}^{2} }}\left( {1 + \frac{\tau }{{U_{b}^{2} }}} \right)} \right]\psi_{s} ,\quad \left( {\tau > 0} \right)$$

for $$\tau > 0$$ and

30 $$\frac{{{\text{d}}^{2} \psi_{s} }}{{{\text{d}}\xi^{2} }} \approx I_{se} \psi_{s}^{2} , \quad \left( {\tau = 0} \right)$$

for $\tau = 0$. The physical solutions for these two approximate equations are

31 $$\psi_{s} = {\text{exp}}\left( {A\xi } \right) ,\quad \left( {\tau > 0} \right)$$

32 $$\psi_{s} = \frac{{A^{{\prime }} }}{{\xi^{2} }} .\quad \left( {\tau = 0} \right)$$

By applying Eqs. (31) and (32) in Eqs. (29) and (30) respectively, the unknown coefficients $A$ and $A^{\prime}$ can be easily find as

$$A = \sqrt {I_{se} } \left[ {1 - \frac{1}{{U_{se}^{2} }}\left( {1 + \frac{\tau }{{U_{se}^{2} }}} \right)} \right]^{1/2} ,\quad A^{{\prime }} = \frac{6}{{I_{se} }} .$$

At a point sufficiently far from the wall, hence, sufficiently near to the plasma-sheath boundary, for example at $\xi = - 100$, $\psi_{s}$ can be determined from (31) and (32) in the warm and cold plasmas respectively. By knowing $\psi_{s}$ and using Eqs. (25) and (22), the values of $I_{s}$ and $U_{s}$ can be found at that point as the boundary conditions.

On the other hand, since the solution $\psi_{s} = \psi_{s} \left( \xi \right)$ is translationally invariant in $\xi$, it is needed to finish the numerical calculations at a proper point. Floating point, where the floating condition $n_{if} v_{xf} = n_{ef} v_{th} /4$ is fulfilled, is selected as the suitable ending point. In the floating condition, subscription ‘*f*’ denotes to the *floating* point and $v_{th} = \sqrt {8k_{B} T_{e} /\pi m_{e} }$ is the electron thermal speed^31^. By using (22) at the floating point, the floating condition in the normalized form is

$$U_{b} = \sqrt {\frac{{m_{i} }}{{2\pi m_{e} }}} {\text{exp}}\left( { - \psi_{f} } \right),$$

33 $$\psi_{f} = \frac{1}{2}{\text{ln}}\left[ {\frac{{m_{i} }}{{2\pi m_{e} \left( {1 + \tau } \right)}}} \right],$$

where $\psi_{f} = \phi_{f} - \phi_{b}$ is the relative electric potential at the floating point. Solving the sheath equations is trivial now.

## Full solution of the eigenvalue problem

Having﻿ examined the both asymptotic limits in the plasma and sheath approaches, we have the full solution of plasma equations in both the body of plasma and near the wall in different approaches. In this section, we discuss about joining these two asymptotic solutions in the same approach smoothly. In other word, we seek to solve the first-order differential equations  (7)–(10) for finite $\epsilon$ by integrating from the plasma center outwards.

It is clear from Eqs. (9) and (10) that derivatives of $I$ and $U$ are undetermined at the regular singularity $U_{r} = \sqrt \tau$ ($U^{\prime}_{r} = 0/0$ and $I^{\prime}_{r} = 0/0$) in which, $\sqrt \tau$ is the normalized ion thermal speed, subscription ‘*r*’ refers to *regular* and the prime denotes differentiation with respect to the spatial coordinate $r$. To remove this ambiguity, the Hopital’s rule obviously can be used here to get

34 $$U_{r}^{{\prime }} = \frac{{S_{r} }}{{R_{r} }} = \frac{{S_{r}^{{\prime }} }}{{R_{r}^{{\prime }} }}{ },$$

or $I_{r}^{{\prime }} = Q_{r} /R_{r} = Q_{r}^{{\prime }} /R_{r}^{{\prime }}$ similarly. It should be noted that Eqs. (5) and (6) result in

35 $$\tau I_{r}^{{\prime }} + I_{r} U_{r} U_{r}^{{\prime }} = H_{r} = U_{r} N_{r} ,$$

which gives rise to

36 $$Q_{r} = S_{r} = R_{r} = 0.$$

Now, using definitions (11), Eqs. (7)–(10), Eq. (36), the following equation

37 $$I^{{\prime }} = \frac{{N - IU^{{\prime }} }}{U},$$

and some algebraic operations, it is straightforward to find,

38 $$S_{r}^{\prime } = \frac{1}{{I_{r} }}\left[ {\frac{1}{{\epsilon^{2} }}\left( {I_{r} - N_{r} } \right)U_{r} I_{r} + \left( {1 + 2\tau } \right)N_{r} F_{r} - \alpha N_{r} U_{r} - F_{r} I_{r} U_{r}^{\prime } } \right],$$

and

39 $$R_{r}^{\prime } = 2U_{r} U_{r}^{\prime } .$$

Finally, applying Eqs. (38) and (39) into Eq. (34) results to the quadratic equation

40 $$aU_{r}^{\prime 2} + bU_{r}^{\prime } + c = 0 .$$

wherein

$$\begin{aligned} & a = 2I_{r} U_{r} ,\quad b = F_{r} I_{r} , \\ & c = \alpha N_{r} U_{r} - \frac{1}{{\epsilon^{2} }}\left( {I_{r} - N_{r} } \right)U_{r} I_{r} - \left( {1 + 2\tau } \right)N_{r} F_{r} . \\ \end{aligned}$$

So,

41 $$U_{r}^{\prime } = \frac{b}{2a}\left( {\sqrt {1 - \frac{4ac}{{b^{2} }}} - 1} \right),$$

is the only reasonable solution of Eq. (40) complying the condition $U_{r}^{\prime } > 0$. Since $a$ and $b$ are two positive constants, the solution is reliable just for $c < 0$. This condition for having physical solutions confines the amounts of $\alpha$, $\epsilon$ and $\tau$ and connects them together. For some proper amounts of $\alpha$, $\epsilon$ and $\tau$, it is easy to determine $U_{r}^{\prime }$ and $I_{r}^{\prime }$ by utilizing Eqs. (41) and (37) respectively.

## Methods and boundary conditions

By removing the ambiguity in $U_{r}^{\prime }$ and $I_{r}^{\prime }$ at the regular point $U_{r}$, the set of differential equations (7)–(10) together with the appropriate boundary conditions should be solved as an eigenvalue problem for $r_{w}$ or the other variables at the wall. In order to find the boundary conditions at the plasma center, the power series expansion of the variables are used. Since $I$ and $\phi$ are even functions and $U$ is an odd function, they can be approximated by

42 $$I_{0} \approx i_{0} + i_{2} r^{2} ,$$

43 $$\phi_{0} \approx \left( {\varphi_{2} + \varphi_{4} r^{2} } \right)r^{2} ,$$

44 $$U_{0} \approx \left( {u_{1} + u_{3} r^{2} } \right)r,$$

and therefore

45 $$F_{0} = \frac{{{\text{d}}\phi }}{{{\text{d}}r}} \approx \left( {2\varphi_{2} + 4\varphi_{4} r^{2} } \right)r,$$

46 $$N_{0} \approx {\text{exp }}\left( { - \phi_{0} } \right),$$

in which, the six unknown factors $i_{0}$, $i_{2}$, $\varphi_{2}$, $\varphi_{4}$, $u_{1}$ and $u_{3}$ should be specified. Using these power series in Eqs. (7)–(10) gives rise to

47 $$i_{0} = 1 + \frac{{\epsilon^{2} \left( {2 + \alpha i_{0} } \right)\left( {i_{0}^{3} + 12\epsilon^{2} } \right)}}{{\left[ {4i_{0}^{3} + \left( {\tau - 3} \right)i_{0}^{2} + \alpha \epsilon^{2} \left( {\tau - 3} \right)i_{0} + 2\left( {4\tau + 3} \right)\epsilon^{2} } \right]i_{0}^{2} }}$$

and

$$\begin{aligned} & u_{1} = \frac{1}{{i_{0} }},\quad \varphi_{2} = \frac{{i_{0} - 1}}{{2\epsilon^{2} }},\quad i_{2} = \frac{{2\varphi_{2} i_{0} - 2u_{1} - \alpha }}{2\tau } \\ & \varphi_{4} = \frac{{i_{2} + \varphi_{2} }}{{12\epsilon^{2} }}, \quad u_{3} = - \frac{{\varphi_{2} + 3i_{2} u_{1} }}{{3i_{0} }} . \\ \end{aligned}$$

By using Eq. (47) iteratively (with $i_{0} = 1$ as initial value) and finding the main factor $i_{0}$, the other coefficients will be explicitly determined^30^.

On the other side, having been defined in “Asymptotic limit of plasma” section, the floating condition is used to end the calculations. This condition in the normalized shape is

$$I_{f} U_{f} = \sqrt {\frac{{m_{i} }}{{2\pi m_{e} }}} {\text{exp}}\left( { - \phi_{f} } \right),$$

48 $$\phi_{f} = \ln \left( {\frac{\beta }{{I_{f} U_{f} }}} \right),$$

with $$\beta = \sqrt {m_{i} /2\pi m_{e} }$$.

Now, set of the equations in $r$ (7)–(10) are integrated by the 6th order Runge Kutta Fehlberg method from the plasma center with the appropriate boundary conditions (42)–(46) until the boundary condition (48) is satisfied. Here, computations have been done for the electropositive gas helium with $\beta \approx 34.18$.

## Results and discussion

**Figure 2:**
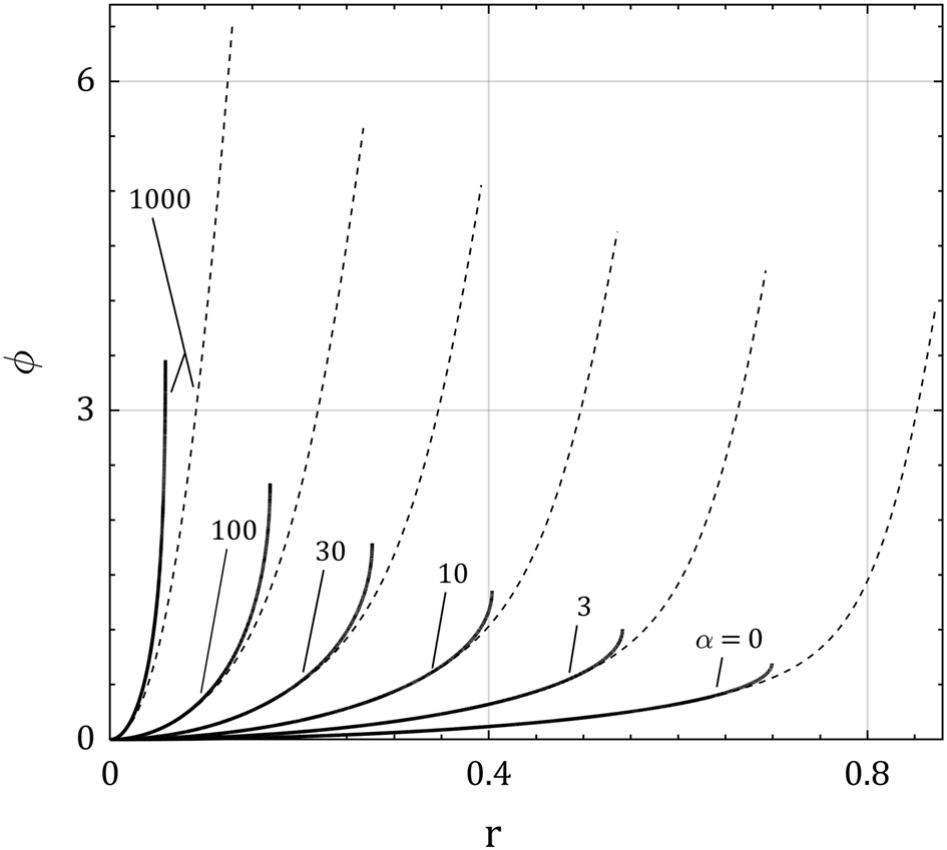
Normalized electric potential distribution $\phi$ for $\tau = 0.5$ in the plasma space $r$. The curves illustrate the plasma asymptotic solution (solid curves for $\epsilon = 0$) and the full solution (dashed curves for $\epsilon = 0.02$) for some values of the collision parameter $\alpha$.

The general form of the plasma asymptotic solution and full solution for the normalized electrical potential $\phi$ is shown in Fig. 2 for $\tau = 0.5$ and different values of $\alpha$. As it is seen, the gradient of the electrical potential tends to infinity by approaching to the plasma-sheath border $r_{b}$ in the plasma asymptotic solution. This figure makes a comparison between the two solutions in the plasma approach for the same plasma parameters. By the results, the effect of the collision parameter is to decrease the boundary layer width (floating width) and to increase the electrical potential at the floating point (floating potential) for fixed values of $\tau$ and $\epsilon$.

**Figure 3:**
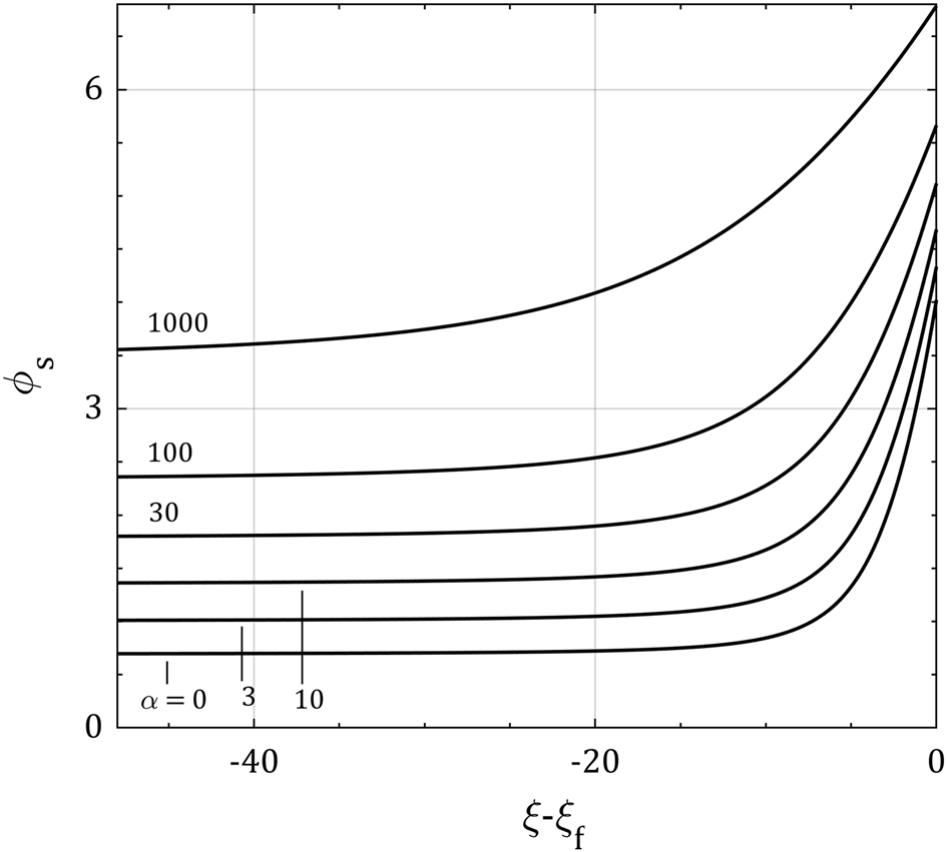
The sheath asymptotic solution for the normalized electric potential distribution $\phi_{s}$ for $\tau = 0.5$ in the sheath space $\xi$. Note how the floating potential $\phi_{f}$ and the potential at the plasma-sheath border $\phi_{b}$ grow up when the collision parameter $\alpha$ increases.

Figure 3 gives the sheath asymptotic solution for the normalized electrical potential $\phi_{s}$ for the same parameters as in Fig. 2 showing how the collision parameter increases the floating potential $\phi_{f}$. It is concluded that increasing the plasma pressure makes the floating electrical potential more negative, therefore reduces the floating current to the wall. By receding from the floating wall, the normalized electrical potential is reduced and saturated to the constant potential $\phi_{b}$ at the plasma-sheath border which is an increasing function of the collision parameter in accordance with Eq. (21-a). Figures 2 and 3 demonstrate the same results in different approaches.

**Figure 4:**
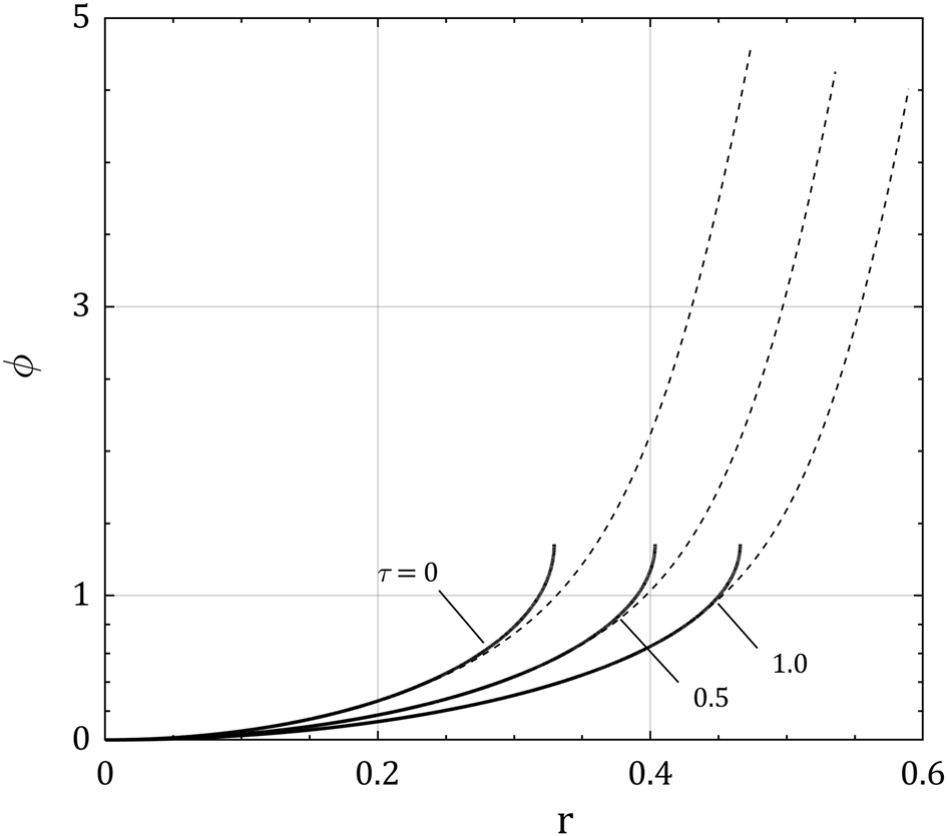
Normalized electric potential distribution $\phi$ for $\alpha = 10$ and $\tau = 0$, 0.5 and 1.0 in plasma space $r$. The curves illustrate the plasma asymptotic solution (solid curves for $\epsilon = 0$) and the full solution (dashed curves for $\epsilon = 0.02$).

Figure 4 represents the plasma asymptotic and full solutions of the normalized electrical potential distribution for $\alpha = 10$ and different values of $\tau$, in which the general form of the electrical potential variations in the both solutions is as in Fig. 2. It is seen that general effect of ion temperature $\tau$ is to enlarge the plasma boundary layer extension and to decrease the normalized floating potential $\phi_{f}$. Similar results can be seen in some recent works on the warm plasma with low ion temperature using approximate methods^27,28,39^.

**Figure 5:**
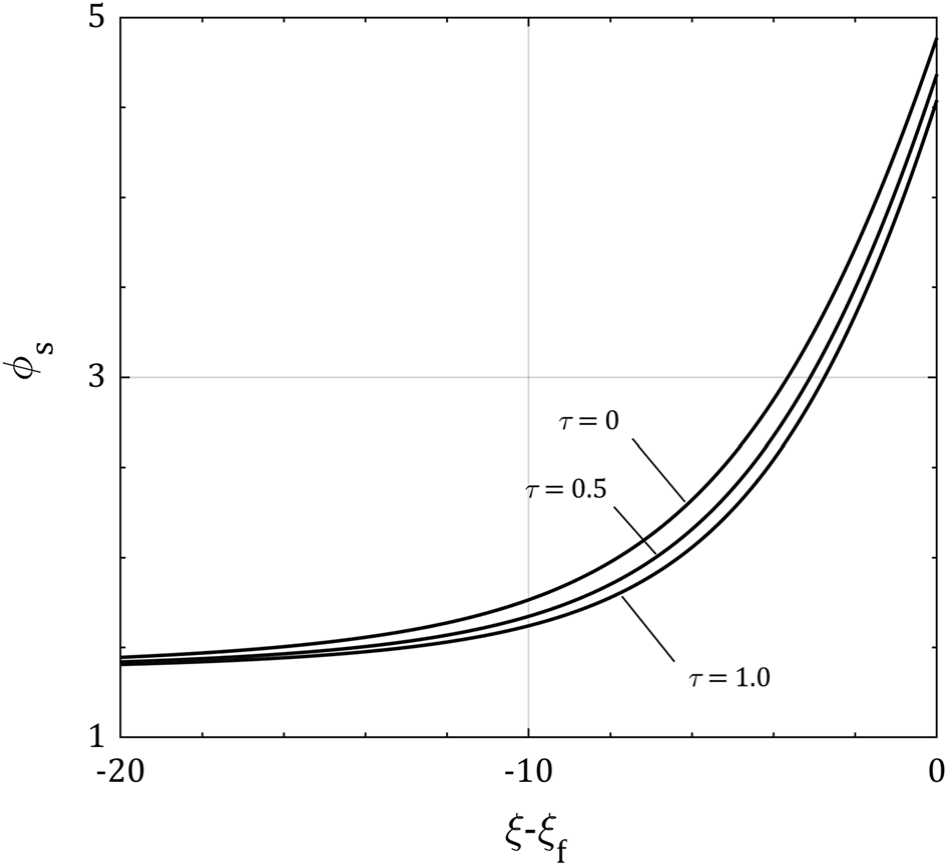
The sheath asymptotic solution for the normalized electric potential distribution $\phi_{s}$ for $\alpha = 10$ and $\tau = 0$, 0.5 and 1.0 in the sheath space $\xi$. Note how the ion temperature $\tau$ decreases the floating potential $\phi_{f}$ and does not affect on the potential at the plasma-sheath border $\phi_{b}$.

Figure 5 depicts the distribution of $\phi_{s}$ and the dependence of $\phi_{f}$ and $\phi_{b}$ on $\tau$ in the sheath approach for the same parameters as in Fig. 3. It can be seen that both of the normalized electrical potential distribution and floating potential are decreasing function of the ion temperature $\tau$. It also can be noted that the ion temperature has no effect on the electrical potential at the plasma-sheath border in accordance with Eq. (21-a)^28^.

**Figure 6:**
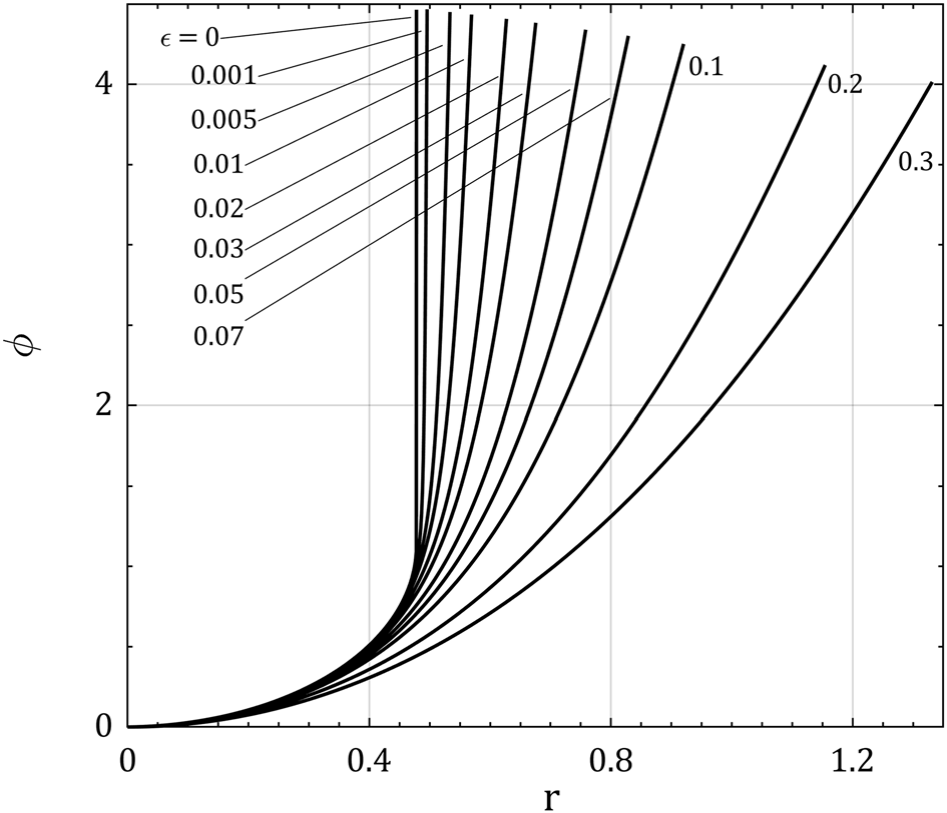
The full solution for the normalized electric potentil distribution $\phi$ in plasma space $r$ for $\tau = 0.5$, $\alpha = 5$ and some values for ionization rate $\epsilon$.

Figure 6 shows the full-solution for the normalized potential profile when the ions isothermally flow towards the wall for $\tau = 0.5$, $\alpha = 5$ and some values for ionization rate $\epsilon$. As can be observed, the effect of $\epsilon$ is to enlarge the floating width and to reduce the normalized floating potential $\phi_{f}$ for a fixed value of $\tau$ and $\alpha$. Indeed, increasing the ionization rate raises the ion density number or the positive space charge which results in growing the electric field and therefore the ion electric drag towards the wall in accordance with the motion of ion and Poisson’s equations. By increasing the ion velocity, the ion density and so the positive space charge will decrease according to continuity equation and this gives rise to decrease the electric field or the slope of electric potential. It can also be realized that the effects of ion temperature and ionization rate are qualitatively the same.

**Figure 7:**
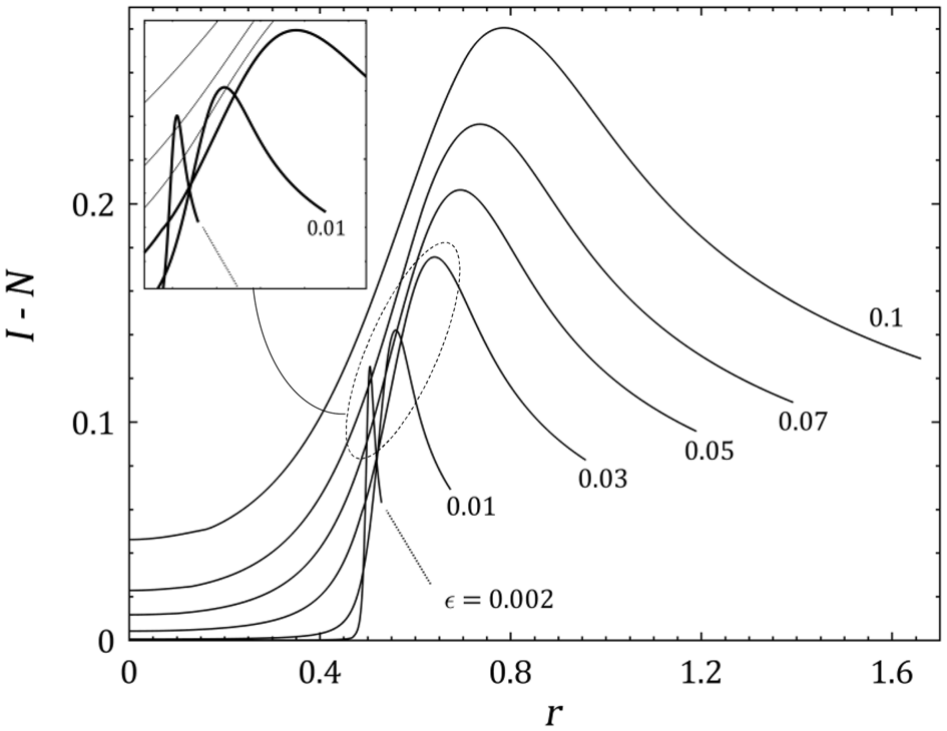
Spatial distribution of the positive space charge $I - N$ in plasma space $r$ for $\tau = 0.5$, $\alpha = 5$, $\phi_{w} = 20$ and some values for the ionization rate $\epsilon$.

Spatial distribution of the normalized positive space charge $I - N$ in the full-solution approach is shown in Fig. 7 as a function of ionization rate $\epsilon$. According to this figure, by receding from the plasma-sheath edge, the positive space charge in the boundary layer increases dramatically and after hitting to a peak begins to fall by approaching to the negative biased wall with $\phi_{w} = 20$. Also, it can be seen that the positive space charge is an ascending and widening function of the ionization rate and it means that the slope of electric potential drops by growing the ionization rate according to the Poisson’s equation. This result is in good consistency with Fig. 6 and as a result, one can say that the electric drag of ions towards the wall will be plummeted by the ionization rate^28^.

Tables 1, 2, 3 and 4 give the floating variables $\phi_{f}$, $U_{f}$, $r_{f}$ and $I_{f}$ respectively, for $\epsilon = 0, 0.002, 0.02$, $\tau = 0.0, 0.1, 0.5, 1.0$ and $\alpha = 0, 0.1, 1, 5, 10, 50, 100, 500$ and $1000$. These tables quote that the floating ion density $I_{f}$, floating ion velocity $U_{f}$ (ion kinetic energy) and floating width $r_{f}$ are decreasing functions, while the floating potential $\phi_{f}$ is an increasing function of the collision parameter $\alpha$ (plasma pressure). In the special case $\epsilon = 0$, it should be noted that $I_{b} /I_{f}$ and therefore $U_{f} = \sqrt {1 + \tau } I_{b} /I_{f}$ is independent of the collision parameter [according to (25) and (33)]. The ion temperature effects is totally the opposite of the collision effects and have already been studied in the collisionless plasmas in^28,29^. In order to complete the discussion, the effects of the ion temperature on the plasma variables in the collisional plasmas has been studied and the results have been summarized in Tables 1, 2, 3 and 4. The results show the weaker effects of ion temperature compared with the effects of ionization and collision parameters.

**Table 1:** Floating electric potential $\phi_{f} \left( {\alpha , \tau , \epsilon } \right)$.

$\epsilon \downarrow$	$\tau \downarrow \alpha \to$	0	0.1	1	5	10	50	100	500	1000
0.0	0	4.2248	4.2398	4.3556	4.6667	4.8870	5.5460	5.8670	6.6471	6.9899
	0.1	4.1771	4.1922	4.3079	4.6191	4.8394	5.4983	5.8193	6.5995	6.9423
	0.5	4.0220	4.0371	4.1528	4.4640	4.6843	5.3432	5.6643	6.4444	6.7872
	1.0	3.8782	3.8933	4.0090	4.3202	4.5404	5.1994	5.5204	6.3005	6.6434
$2 \times 10^{ - 3}$	0	4.2047	4.2200	4.3370	4.6502	4.8716	5.5325	5.8531	6.6273	6.9630
	0.1	4.1583	4.1737	4.2901	4.6034	4.8248	5.4861	5.8074	6.5828	6.9199
	0.5	4.0159	4.0315	4.1486	4.4622	4.6822	5.3395	5.6616	6.4298	6.7696
	1.0	3.8704	3.8858	4.0034	4.3120	4.5337	5.1892	5.5176	6.2864	6.6264
$2 \times 10^{ - 2}$	0	4.1223	4.1379	4.2583	4.5785	4.8021	5.4506	5.7509	6.4204	6.6840
	0.1	4.0806	4.0966	4.2169	4.5374	4.7611	5.4096	5.7101	6.3879	6.6380
	0.5	3.9524	3.9680	4.0884	4.4073	4.6282	5.2706	5.5737	6.2731	6.5589
	1.0	3.8425	3.8591	3.9773	4.2888	4.5040	5.1396	5.4432	6.1498	6.4433

**Table 2:** Floating ion velocity $U_{f} \left( {\alpha , \tau , \epsilon } \right)$.

$\epsilon \downarrow$	$\tau \downarrow \alpha \to$	0	0.1	1	5	10	50	100	500	1000
0.0	0	2.8396	2.8396	2.8396	2.8396	2.8396	2.8396	2.8396	2.8396	2.8396
	0.1	2.8757	2.8757	2.8757	2.8757	2.8757	2.8757	2.8757	2.8757	2.8757
	0.5	3.0095	3.0095	3.0095	3.0095	3.0095	3.0095	3.0095	3.0095	3.0095
	1.0	3.1587	3.1587	3.1587	3.1587	3.1587	3.1587	3.1587	3.1587	3.1587
2 × 10^−3^	0	2.8222	2.8208	2.8078	2.7499	2.6100	2.2240	1.8306	0.7601	0.4598
	0.1	2.8580	2.8566	2.8432	2.7847	2.7140	2.2533	1.8555	0.7689	0.4781
	0.5	2.9902	2.9888	2.9748	2.9136	2.8398	2.3628	1.9483	0.9023	0.5911
	1.0	3.1379	3.1362	3.1216	3.0575	2.9808	2.4822	2.0492	1.0163	0.7119
2 × 10^−2^	0	2.7510	2.7419	2.6638	2.3657	2.0749	1.0383	0.6543	0.2004	0.1193
	0.1	2.7850	2.7760	2.6970	2.3958	2.1016	1.0514	0.6599	0.2129	0.1316
	0.5	2.9113	2.9018	2.8197	2.5071	2.2010	1.0748	0.8005	0.2659	0.1575
	1.0	3.0529	3.0433	2.9568	2.6304	2.3102	1.3172	0.9479	0.3300	0.1965

**Table 3:** Floating width $r_{f} \left( {\alpha , \tau , \epsilon } \right)$.

$\epsilon \downarrow$	$\tau \downarrow \alpha \to$	0	0.1	1	5	10	50	100	500	1000
0.0	0	0.5708	0.5639	0.5133	0.3969	0.3294	0.1848	0.1380	0.0663	0.0477
	0.1	0.5987	0.5914	0.5383	0.4163	0.3455	0.1938	0.1447	0.0696	0.0500
	0.5	0.6991	0.6906	0.6286	0.4861	0.4035	0.2263	0.1690	0.0812	0.0584
	1.0	0.8072	0.7975	0.7259	0.5613	0.4659	0.2613	0.1951	0.0938	0.0675
2 × 10^−3^	0	0.6014	0.5944	0.5431	0.4253	0.3569	0.2088	0.1599	0.0829	0.0627
	0.1	0.6288	0.6214	0.5677	0.4441	0.3724	0.2172	0.1659	0.0860	0.0650
	0.5	0.7221	0.7134	0.6505	0.5066	0.4239	0.2460	0.1878	0.0977	0.0736
	1.0	0.8159	0.8060	0.7335	0.5696	0.4761	0.2763	0.2112	0.1100	0.0828
2 × 10^−2^	0	0.7530	0.7451	0.6876	0.5536	0.4739	0.2948	0.2350	0.1421	0.1165
	0.1	0.7778	0.7697	0.7098	0.5702	0.4871	0.3021	0.2414	0.1459	0.1209
	0.5	0.8642	0.8548	0.7858	0.6270	0.5347	0.3342	0.2675	0.1597	0.1295
	1.0	0.9448	0.9341	0.8567	0.6836	0.5845	0.3686	0.2948	0.1749	0.1413

**Table 4:** Floating ion density $I_{f} \left( {\alpha , \tau , \epsilon } \right)$.

$\epsilon \downarrow$	$\tau \downarrow \alpha \to$	0	0.1	1	5	10	50	100	500	1000
0.0	0	0.1761	0.1735	0.1545	0.1132	0.0908	0.0470	0.0341	0.0156	0.0111
	0.1	0.1824	0.1796	0.1600	0.1172	0.0940	0.0487	0.0353	0.0162	0.0115
	0.5	0.1999	0.2004	0.1785	0.1308	0.1049	0.0543	0.0394	0.0181	0.0128
	1.0	0.2204	0.2201	0.1964	0.1439	0.1154	0.0597	0.0433	0.0199	0.0141
2 × 10^−3^	0	0.1808	0.1781	0.1593	0.1189	0.0978	0.0609	0.0536	0.0515	0.0501
	0.1	0.1870	0.1842	0.1648	0.1230	0.1011	0.0629	0.0554	0.0526	0.0508
	0.5	0.2061	0.2030	0.1814	0.1354	0.1114	0.0695	0.0611	0.0601	0.0586
	1.0	0.2226	0.2194	0.1959	0.1465	0.1208	0.0757	0.0669	0.0624	0.0606
2 × 10^−2^	0	0.2014	0.1989	0.1815	0.1484	0.1253	0.1014	0.0715	0.0657	0.0612
	0.1	0.2074	0.2048	0.1869	0.1527	0.1392	0.1055	0.0789	0.0708	0.0654
	0.5	0.2257	0.2228	0.2032	0.1662	0.1518	0.1135	0.0885	0.0792	0.0711
	1.0	0.2401	0.2371	0.2167	0.1784	0.1637	0.1321	0.1043	0.0836	0.0783

## Summary and conclusions

This paper expands a numerical-analytical method to describe the boundary layer of collisional warm plasmas in touch with the conducting planar surface when the ion temperature is comparable to the electron temperature and the plasma pressure is sufficiently high such that the ion-neutral elastic collision in the presheath must be taken into account. Ions are created by electron impact ionization in the active plasma that allows the plasma boundary layer to be formed.

Considering the effects of space-charge, the mathematical description of plasmas boundary layer by means of the hydrodynamic or fluidal equations leads to a singular point when the mean velocity of ions reaches the ion thermal speed $U_{r} = \sqrt \tau$ ($c_{th} = \sqrt {k_{B} T_{i} /m_{i} }$). Starting with the ion continuity and momentum transfer equations including terms of collision, pressure and inertia, the positive column of thermal plasmas at high pressure is examined. By removing the singularity and finding smooth solutions, the essential smoothing conditions around the singular point within the boundary layer of plasma is investigated which leads to a well-defined eigenvalue problem.

The electrical potential distribution in the plasma boundary layer, has been shown in three approaches; plasma and sheath asymptotic solutions for $\epsilon = 0$ and full solution for $\epsilon \, \gtrsim \,0$. While the ion temperature and smallness parameter decrease the electric potential profile, the ion-neutral collision increases it in these three approaches. Also, the global floating variables including the ion velocity, ion density, electrical potential, boundary layer width, and how they are all affected by the plasma parameters (ion temperature, collision and smallness parameters) have been presented. All these consequences are much more pronounced with collision and smallness parameters than with the ion temperature.
